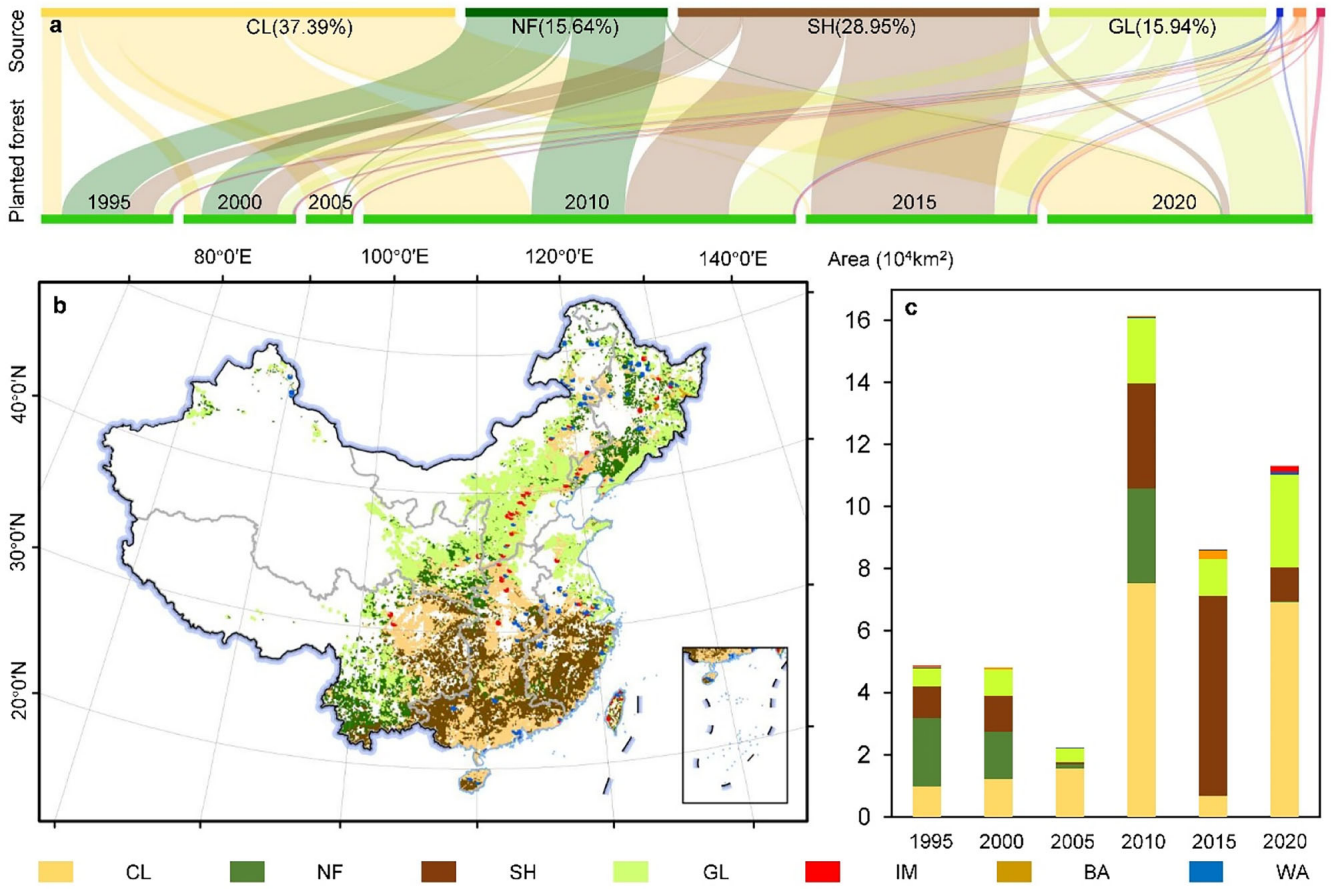# Author Correction: Carbon storage through China’s planted forest expansion

**DOI:** 10.1038/s41467-025-64762-8

**Published:** 2025-10-10

**Authors:** Kai Cheng, Haitao Yang, Shengli Tao, Yanjun Su, Hongcan Guan, Yu Ren, Tianyu Hu, Wenkai Li, Guangcai Xu, Mengxi Chen, Xiancheng Lu, Zekun Yang, Yanhong Tang, Keping Ma, Jingyun Fang, Qinghua Guo

**Affiliations:** 1 Institute of Remote Sensing and Geographic Information System, School of Earth and Space Sciences, Peking University, Beijing, 100871 China; 2 Institute of Ecology, College of Urban and Environmental Sciences, Peking University, Beijing, 100871 China; 3 State Key Laboratory of Vegetation and Environmental Change, Institute of Botany, Chinese Academy of Sciences, Beijing, 100093 China; 4 University of Chinese Academy of Sciences, Beijing, 100049 China; 5 School of Tropical Agriculture and Forestry, Hainan University, Haikou, 571737 China; 6 School of Geography and Planning, Sun Yat-Sen University, Guangzhou, 510275 China; 7 Beijing GreenValleyTechnology Co. Ltd, Beijing, 100091 China; 8 Key Laboratory for Earth Surface Processes of the Ministry of Education, Peking University, Beijing, 100871 China

**Keywords:** Climate-change mitigation, Forestry, Forestry, Sustainability, Biogeography

Correction to: *Nature Communications* 10.1038/s41467-024-48546-0, published online 15 May 2024

In Figures 1, 2, and 4, the errors of longitude and latitude annotations occurred. Please see the details as following figures:

**Original Figure 1 The latitude markings in Figure 1 are incorrect**.
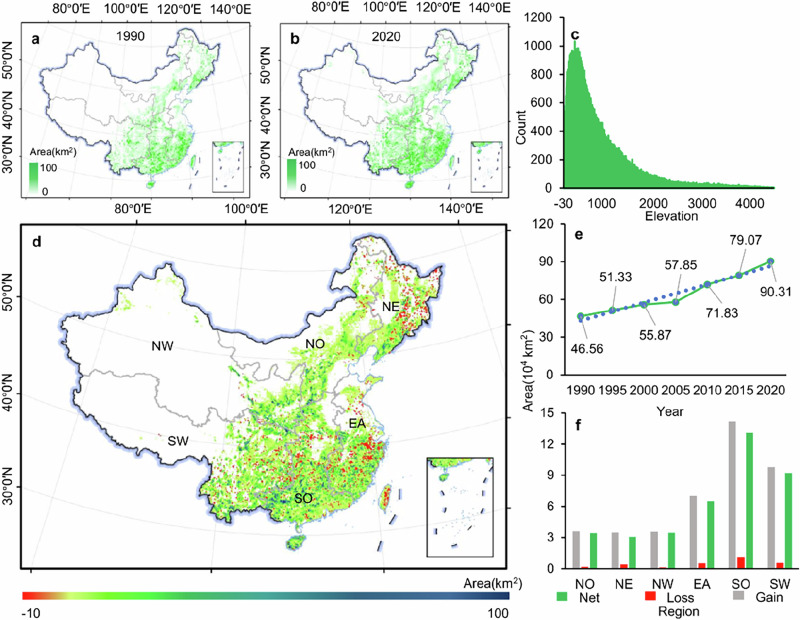


**Revised Figure 1**.
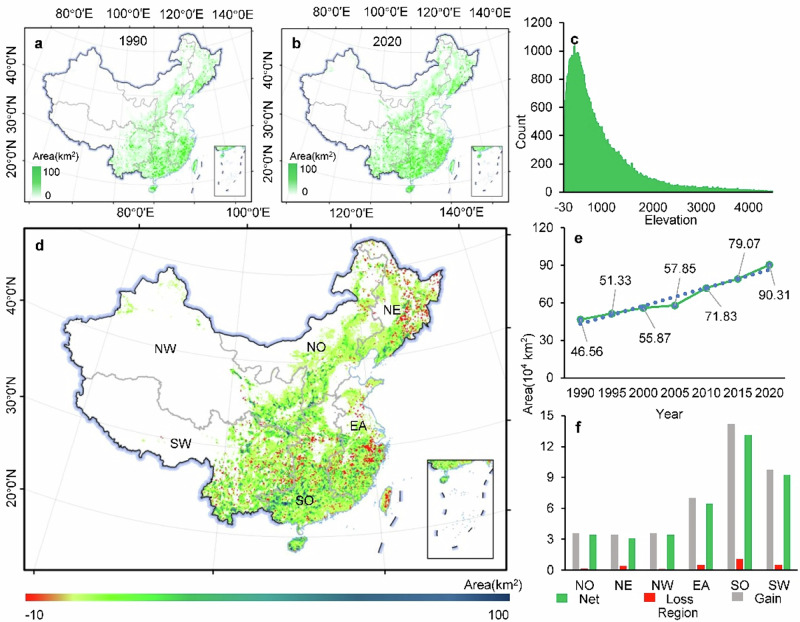


**Original Figure 2 The latitude markings in Figure 2 are incorrect**.
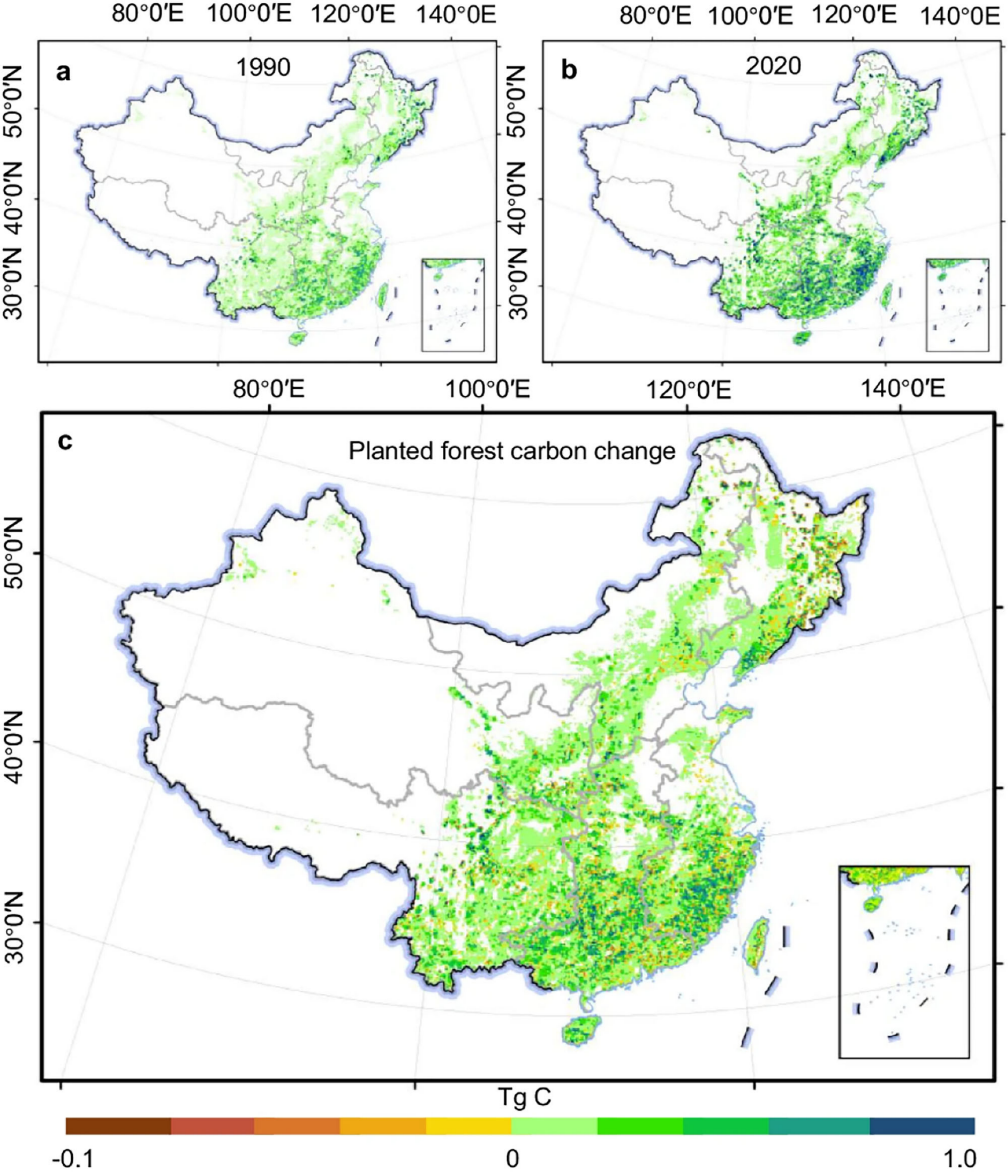


**Revised Figure 2**.
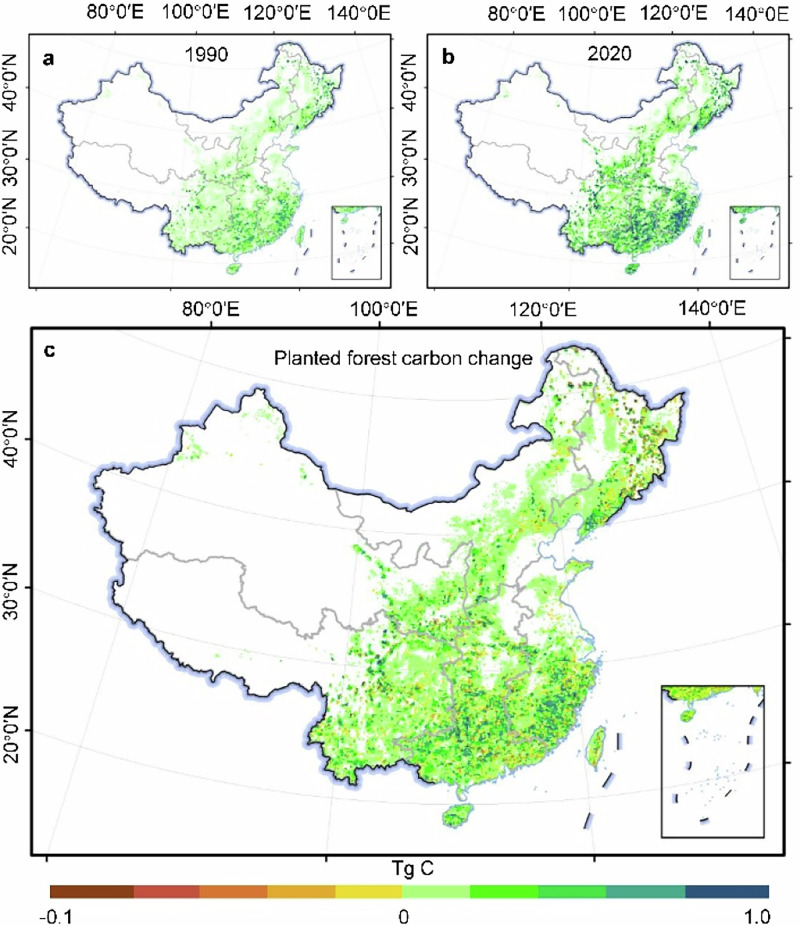


**Original Figure 4 The latitude and longitude markings in Figure 4 are incorrect**.
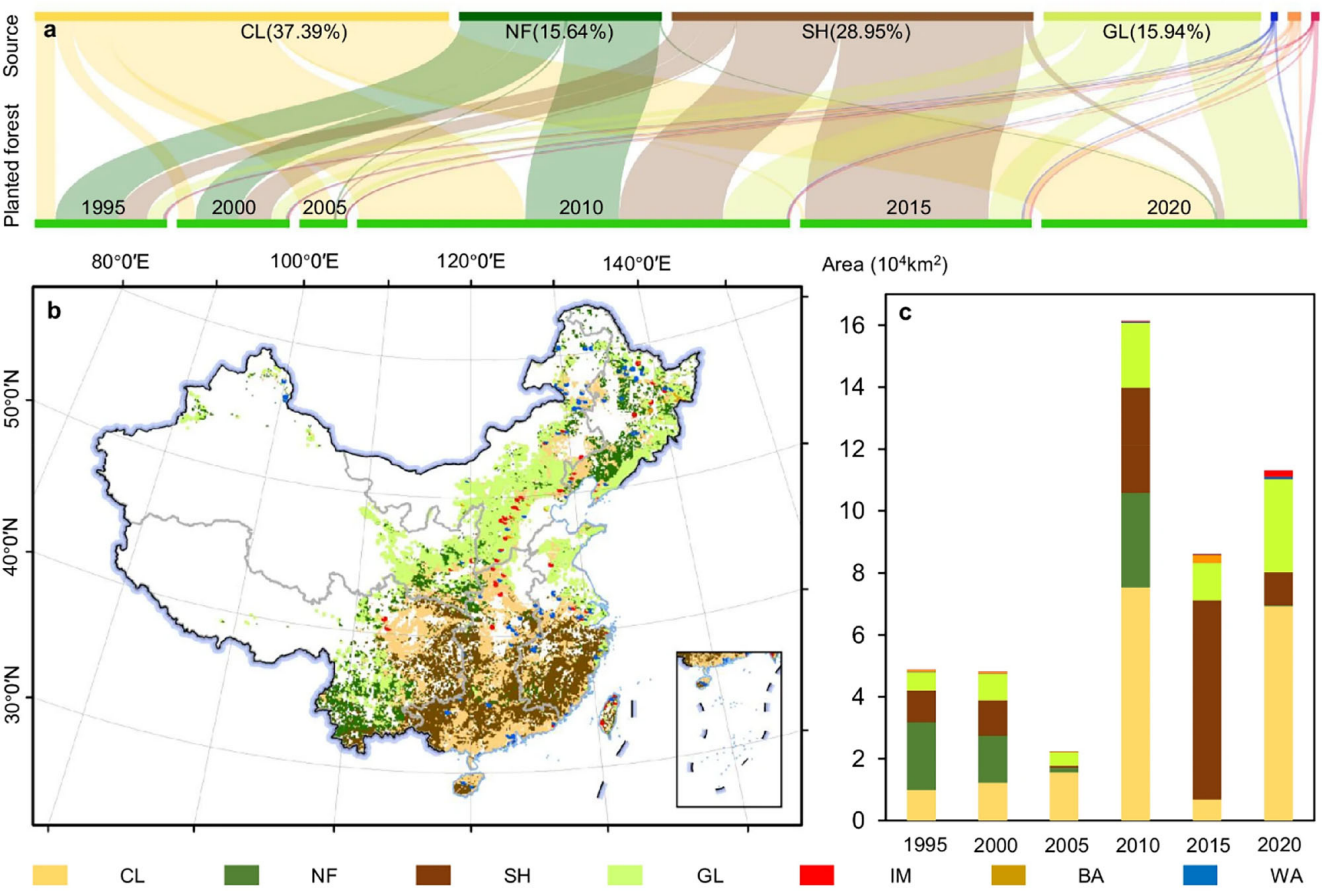


**Revised Figure 4**.