# Supplementary material for: Carbon storage through China’s planted forest expansion
Source: Nat Commun. 2024 May 15;15:4106. doi: 10.1038/s41467-024-48546-0 (PMC11096308; doi:10.1038/s41467-024-48546-0)
Supplement: Supplementary file 1 — Supplementary Information [file 41467_2024_48546_MOESM1_ESM.pdf]

# Supplementary Information for

**Harnessing the expansion of China's planted forest for carbon storage**

**Authors: Kai Cheng[1, 2#], Haitao Yang[1#], Shengli Tao[2], Yanjun Su[3, 4], Hongcan Guan[5], Yu Ren[1, 2], Tianyu Hu[3, 4], Wenkai Li[6], Guangcai Xu[7], Mengxi Chen[1], Xiancheng Lu[1], Zekun Yang[1], Yanhong Tang[2], Keping Ma[3, 4], Jingyun Fang[2, 8], Qinghua Guo[1, 2*]**

**Corresponding author. Email: guo.qinghua@pku.edu.cn**

**This file includes:**

**Supplementary Note 1-5**

**Supplementary Figures 1-19**

**Supplementary Tables 1-5**

**Supplementary Reference**

# Supplementary Note 1 | Forest masks

Forest masks were selected according to three existing time-series land use/land cover (LULC) datasets covering China: the global LULC product with a fine classification system at 30 m (GLC_FCS30) [1], China's LULC dataset (CLCD) [2], and China's land use/cover datasets (CLUDs) and all documented detailed forest types in China for 1990, 1995, 2000, 2005, 2010, 2015, and 2020 (Supplementary Fig. 18). To choose a reliable forest mask, we first evaluated the three LULC datasets using our massive field samples (over 170,000). CLCD yielded the highest overall accuracy during each period (Supplementary Fig. 19). Therefore, we selected the forest regions from the CLCD dataset as the forest mask and assumed that they represented the accurate forest extent for each period. Our research was conducted using these forest masks.

GLC_FCS30 LULC data were downloaded from the Data Sharing and Service Portal (available at https://data.casearth.cn/en/). CLCD LULC data were downloaded from the website of https://zenodo.org/record/5816591#.Y9tkpnBBztU. CLUD LULC data were collected from the Resource and Environment Science and Data Center (available at https://www.resdc.cn/ ).

## Supplementary Note 2 | Definitions of vegetation, temporal and textural indices.

The vegetation indices used in this work included the bare soil index (BSI), enhanced vegetation index (EVI), normalized difference vegetation index (NDVI), modified soil-adjusted vegetation index (MSAVI), and soil-adjusted vegetation index (SAVI). They were calculated using the equations below:

$$\mathrm{BSI} = \frac{(\rho_{SWIR1} + \rho_{red}) - (\rho_{NIR} + \rho_{blue})}{(\rho_{SWIR1} + \rho_{red}) + (\rho_{NIR} + \rho_{blue})} \tag{1}$$

$$\mathrm{EVI} = 2.5 \times \frac{\rho_{NIR} - \rho_{red}}{\rho_{NIR} + 6 \times \rho_{red} - 7.5 \times \rho_{blue} + 1} \tag{2}$$

$$\mathrm{NDVI} = \frac{\rho_{NIR} - \rho_{red}}{\rho_{NIR} + \rho_{red}} \tag{3}$$

$$\mathrm{MSAVI} = \frac{2 \times \rho_{NIR} + 1 - sqrt\big((2 \times \rho_{NIR} + 1)^2 - 8 \times (\rho_{NIR} - \rho_{red})\big)}{2} \tag{4}$$

$$\mathrm{SAVI} = \frac{(\rho_{NIR} - \rho_{red}) \times 2}{\rho_{NIR} + \rho_{red} + 1} \tag{5}$$

where $\rho_{blue}$, $\rho_{red}$, $\rho_{NIR}$, and $\rho_{SWIR}$ are the surface reflectance values of Blue, Red, near-infrared, and shortwave infrared of the Landsat images.

Temporal features were extracted through analysis of the 1990-2020 NDVI and EVI time-series data derived from Landsat 5 TM Collection 1 Tier 1 8-Day NDVI/EVI composite, Landsat 7 Collection 1 Tier 1 8-Day NDVI/EVI composite, and Landsat 8 Collection 1 Tier 1 8-Day NDVI/EVI composite imagery. Harmonic analysis, a popular reconstruction method, was applied using the equation below to reduce the random noise of the NDVI/EVI time series and ensure the reliability of the extracted temporal features [3]. Then, temporal features, including amplitude, phase, magnitudes of the fitted time series, and the root mean squared error (RMSE) between the original

and fitted values, were all extracted as the temporal feature set.

$$\tilde{y}(t_j) = a_0 + \sum_{i=1}^{m}[a_i \cos(2\pi f_i t_j) + b_i \sin(2\pi f_i t_j)] \quad (6)$$

where $\tilde{y}$ is the reconstructed NDVI time series, $m$ is the number of harmonics and set to 1 in this study. $t_j$ is the time when the original value of $\tilde{y}$ was observed, where $j=1, 2, \ldots, N$ with $N$ as the maximum number of observations in a time series. $a_i$, $b_i$ are the coefficients of the trigonometric components with frequencies $f_i$, and $a_0$ is the coefficient when the frequency is zero.

Textural features were calculated using the gray-level co-occurrence matrix approach, which is widely used in textural information extraction in remote sensing classification [4-6]. This method can be easily implemented in google earth engine (GEE) platform and enables the extraction of 18 textural indices for the input band [7]. The textural information between natural forests and planted forests showed considerable differences in the remote sensing images [8]. All 18 textural indices of each spectral band and vegetation indices were retained as the feature library (Supplementary Table 5). Each textural index is explained in detail on the website (https://developers.google.com/earth-engine/apidocs/ee-image-glcmtexture ).

As terrain also influences the distribution of planted forests [9,10], elevation, aspect, and slope features were derived according to the digital elevation model (DEM) data of the Shuttle Radar Topography Mission in GEE. In total, 220 features were constructed

for mapping planted forests (Supplementary Table 5).

## Supplementary Note 3 | Mapping planted forests

Given the computation resource limitation of GEE, we divided China into six geographic zones and implemented mapping at regional scales. The random forest (RF) classifier was used to map planted forests owing to its ability to handle high-dimensional data, tolerance to sample errors, and robustness to missing data [2,11]. After tests were conducted with different numbers of trees, from 10 to 500, the number of trees was set to 100, according to the exported overall classification accuracies.

To reduce the number of features used in the classification process and economize computational resources, we used the recursive feature elimination with cross-validation (RFE-CV) feature selection approach on a local computer to construct the optimal feature set for each region and period [12]. This approach involves searching for a subset of features, starting with all features in the training dataset and eliminating features that negatively influence accuracy through cross-validation. RFE-CV was implemented using Python 3.8 on a local computer, with the k-fold parameter in CV set to 5. The optimal number of features and selected features for each region were determined according to the output standard deviation of accuracy (Supplementary Fig. 12 and Table 4). Using the constructed feature set for each region and period, we implemented planted forest mapping using RF and the training data on the GEE cloud-processing platform.

## Supplementary Note 4 | Accuracy assessment of planted forest maps

The accuracy of the planted forest maps was assessed using four data sources: (1) the validation data from field surveys (35,074 samples), (2) the validation data from stratified random sampling, (3) the statistical data of China's planted forest resource inventory between 2000 and 2020 for each province, and (4) intercomparison with forest inventory map. The validation data from field surveys and stratified random sampling were used to construct a confusion matrix, in which the overall accuracy (OA), F1 score, producer's accuracy (PA), and user's accuracy (UA) were calculated using the following equations to assess the accuracies.

$$\mathrm{OA} = \frac{Num_{correct}}{Sum} \quad (7)$$

$$\mathrm{F1} = \frac{1}{\frac{1}{2}(\frac{1}{PA} + \frac{1}{UA})} = \frac{2PA \cdot UA}{PA + UA} \quad (8)$$

where $Num_{correct}$ is the number of testing samples mapped correctly, $Sum$ is the total number of testing samples, PA is the producer's accuracy, and UA is the user's accuracy. The macro-average of the F1 score is the average F1 value for all classes. During the calculation of the average F1 value, all classes are assigned equal weights, and the actual sample frequency of occurrence is ignored.

1. Validation data from field surveys

A total of 35,074 field samples were used to validate our planted forest maps (Supplementary Fig. 12b). The evaluation results (Supplementary Fig. 14) indicated

that the OA of the resultant maps ranged from 78.4% to 82.7% (Supplementary Fig. 4a). The UA (a measure of commission error) for planted forests was between 78.0% and 83.0% (Supplementary Fig. 4a). The PA (a measure of omission error) ranged from 78.0% to 88.0%. The F1 score (a balance of UA and PA) ranged from 0.88 to 0.93 (Supplementary Fig. 4a). Accuracy increased with time, with the planted forest map for 2020 yielding the highest accuracy.

## 2. Validation data from systematic random sampling

The systematic random sampling approach, which is regarded as one of the most robust methods for assessing the accuracy of LULC maps in remote sensing, was also applied to validate the resultant maps [13,14]. We first generated a grid with a resolution of 0.1°×0.1° in China's forest regions to collect samples for accuracy assessment. For each grid, one point was randomly generated, resulting in the generation of 15,578 random points (Supplementary Fig. 13). Each point was interpreted using the planted forest inventory map and google earth high resolution images by vegetation ecological experts. The systematic random validation samples for each map were generated and used to calculate the OA, F1, PA, and UA. The validation results revealed that the OA and F1 scores of the planted forest map for 2020 were 81.8% and 0.9, respectively, while the UA and PA of planted forests were 82.0% and 90.0% (Supplementary Fig. 4b), demonstrating the high reliability of the planted forest dataset for revealing the spatiotemporal dynamics of planted forests.

3. Statistical data of China’s planted forest resource inventory

We collected the 2000-2020 statistical data of planted forests for each province from the China forestry yearbook, which recorded the planted forest areas. Then, the planted forest areas in our dataset for each province were calculated. The planted forest areas from our dataset agreed well with those from the statistical data (Supplementary Fig. 3). The $R^2$ between our mapped areas and statistical data ranged from 0.8 to 0.9. Among the maps for the different years, the planted forest area for 2000 featured the best agreement with the forest inventory data ($R^2 = 0.87$), followed by the map for 2010 ($R^2 = 0.85$) (Supplementary Fig. 3). This further demonstrates the good performance of our method and the reliability of the resultant dataset we produced.

4. Available planted forest maps

Peng et al. [15] obtained the only existing planted forest map by digitizing the 7th National Forest Inventory (2004-2008). We compared this map with our results for 2000, 2005, and 2010 via spatial analysis within a 0.1° grid (Supplementary Fig. 19). The area difference between each grid was mostly in the range of 0 to 50 km$^2$ (Supplementary Fig.19), suggesting that the dataset was reliable for depicting China’s planted forests.

## Supplementary Note 5 | Pixel uncertainty analysis

Pixel uncertainty analysis is required and important in LULC mapping based on remote sensing images [16], as it can reveal the confidence of the dataset in different regions. According to the output classification probability of each pixel in the random forest classifier, we generated the uncertainty maps (Supplementary Fig. 16) for the years 1990, 1995, 2000, 2005, 2010, 2015, and 2020 using the equation below. The analysis showed that low uncertainties (< 0.3) were present in large areas of the dataset (65.71%-80.06%), particularly in the south and east regions (Supplementary Fig. 16). Pixel uncertainty ($U_{pixel}$) was calculated as follows:

$$U_{pixel} = 1 - P_{max} \tag{11}$$

where $P_{max}$ is the maximum probability of a pixel being classified into planted forest [17].

**Supplementary Fig. 1 | Spatial distribution maps of China's planted forests in 1990, 1995, 2000, 2005, 2010, 2015 and 2020.**

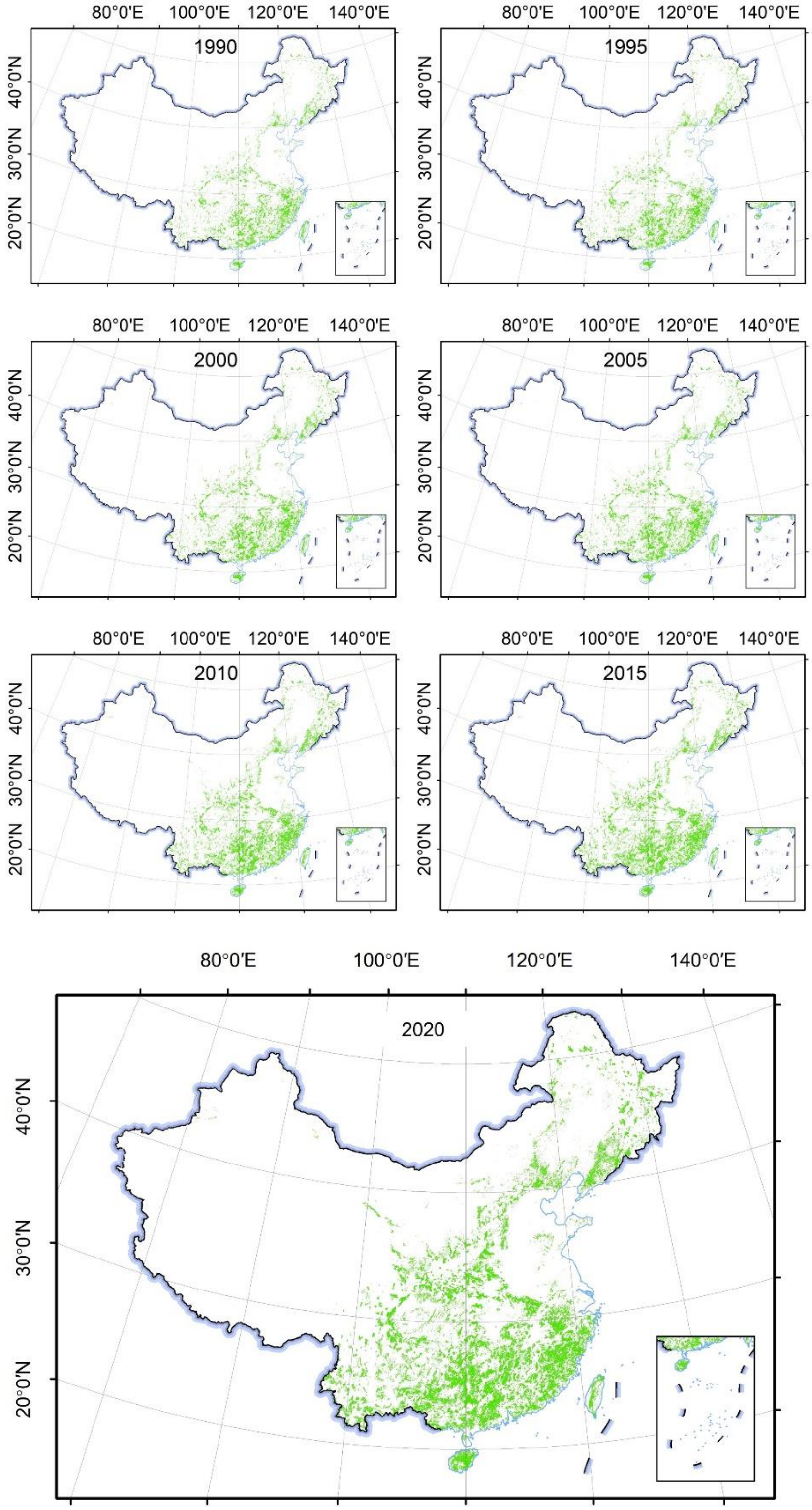

**Supplementary Fig. 2 | Comparison of planted forest area within each 0.1° between the resultant maps and available planted forest dataset in 2000, 2010, and 2020.** a, b, and c are the spatial distribution of area differences; d is the frequency statistics of grid counts with various area differences.

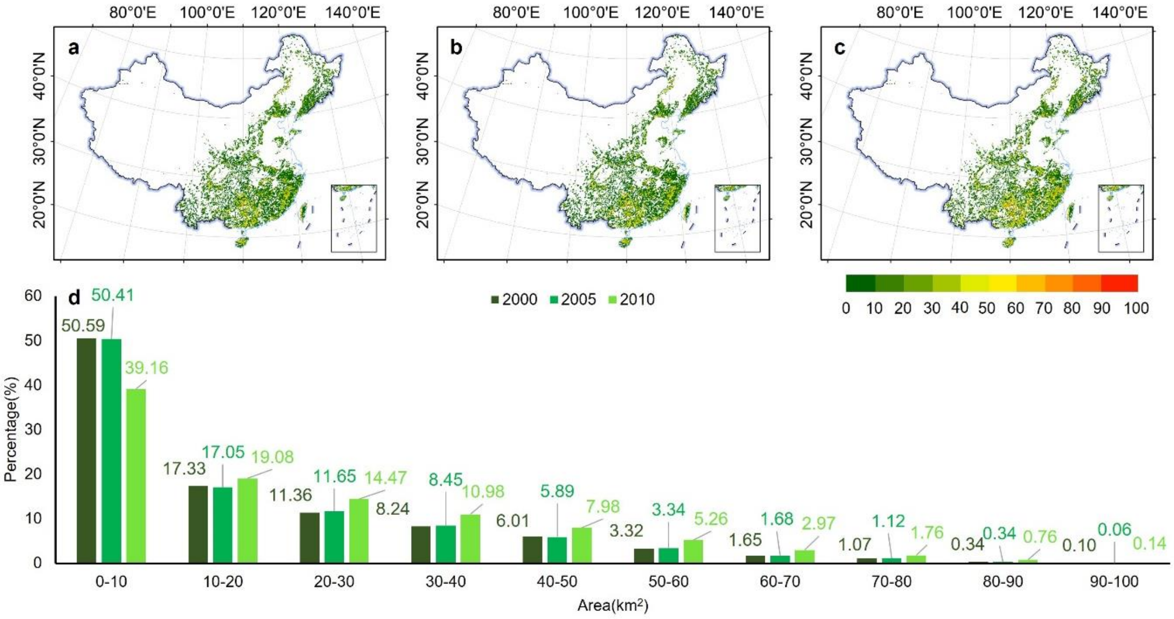

**Supplementary Fig. 3 | Comparison of planted forest area between the resultant wall-to-wall map and statistics of National Forest Inventory for the year 2000, 2005, 2010, 2015, and 2020 at provincial scale.**

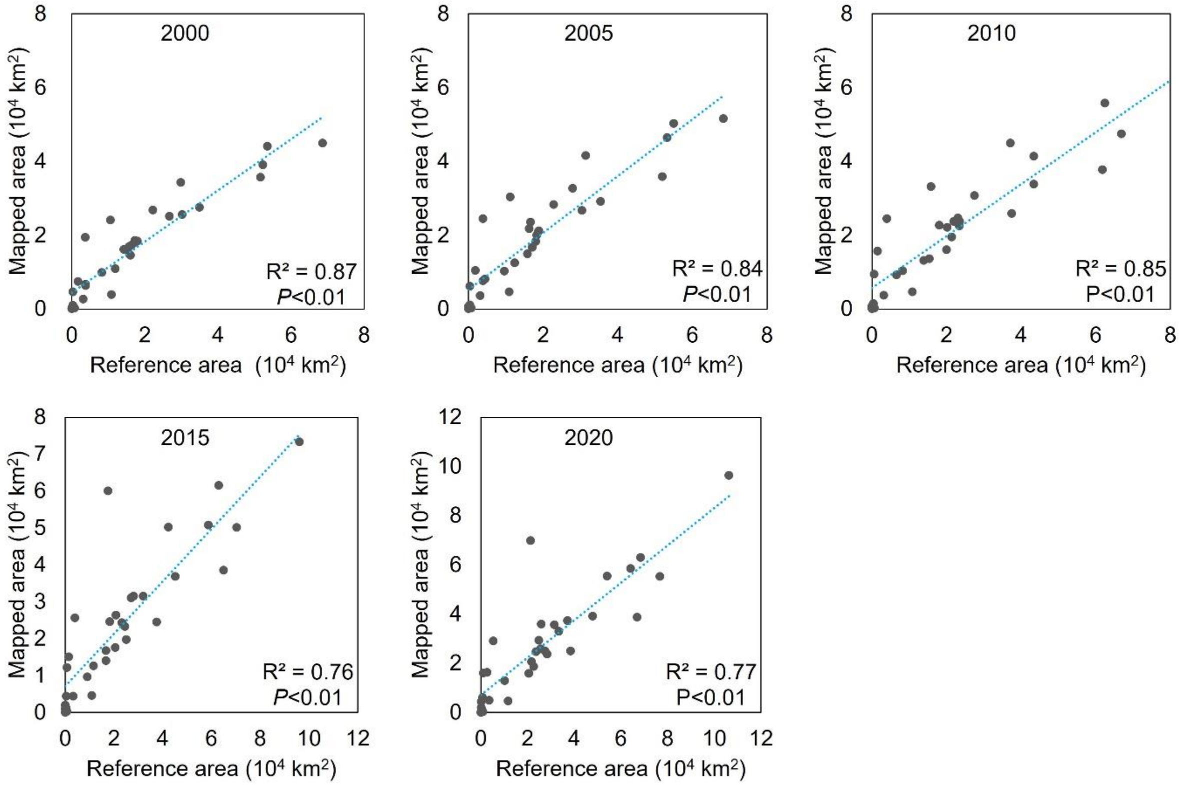

**Supplementary Fig. 4 | Results of Confusion matrix. (a)** based on field validations; **(b)** based on stratified random samples. PF: planted forests; NF: natural forests.

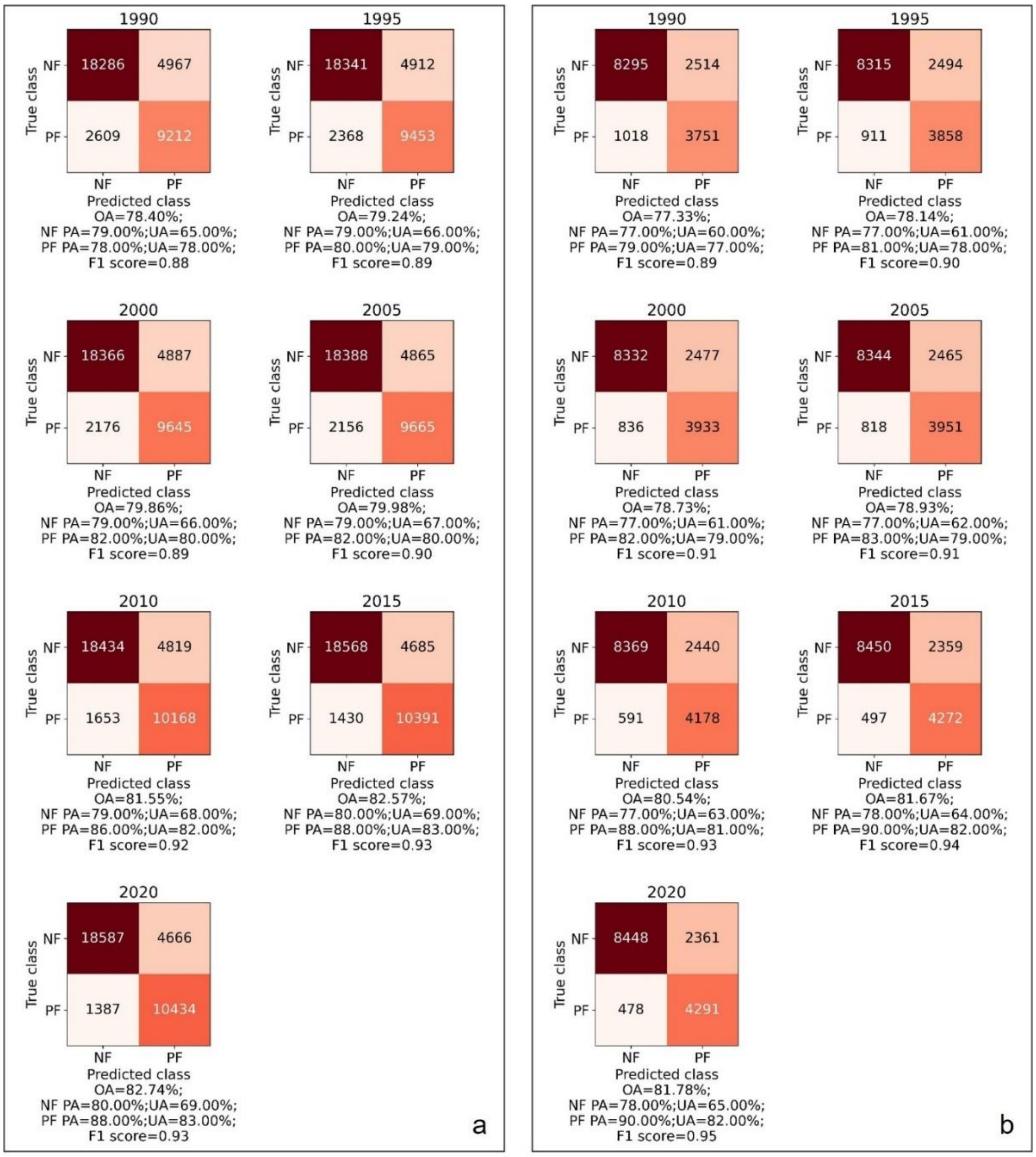

**Supplementary Fig. 5 | Planted forest area in 2020 at regional and provincial scales. (a)** planted forest area in NE, NO, NW, EA, SO and SW; (**b)** planted forest area in each province in China**.** NE: northeast, NO: north, NW: northwest, EA: east, SO: south, SW: southwest.

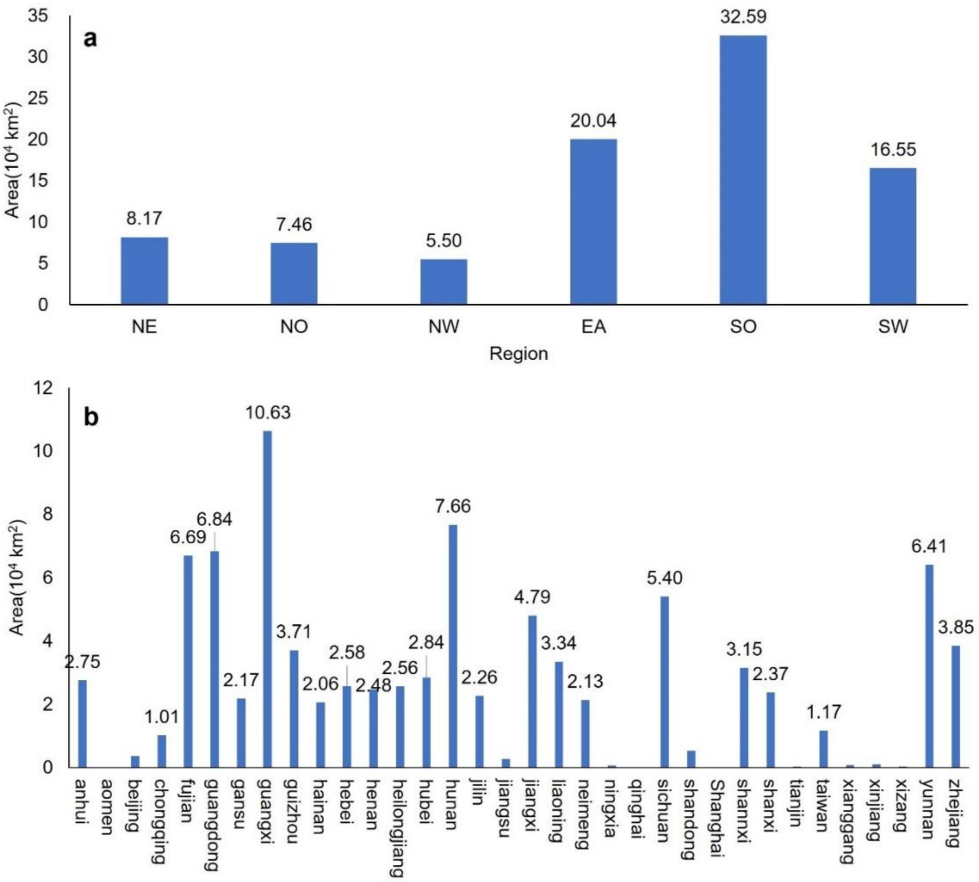

**Supplementary Fig. 6 | Temporal dynamic change trend of planted forest area and net increase area from 1990 to 2020 at the regional scale**. **(a)** planted forest area change trend; **(b)** net increase area change trend of planted forest area. NE: northeast, NO: north, NW: northwest, EA: east, SO: south, SW: southwest.

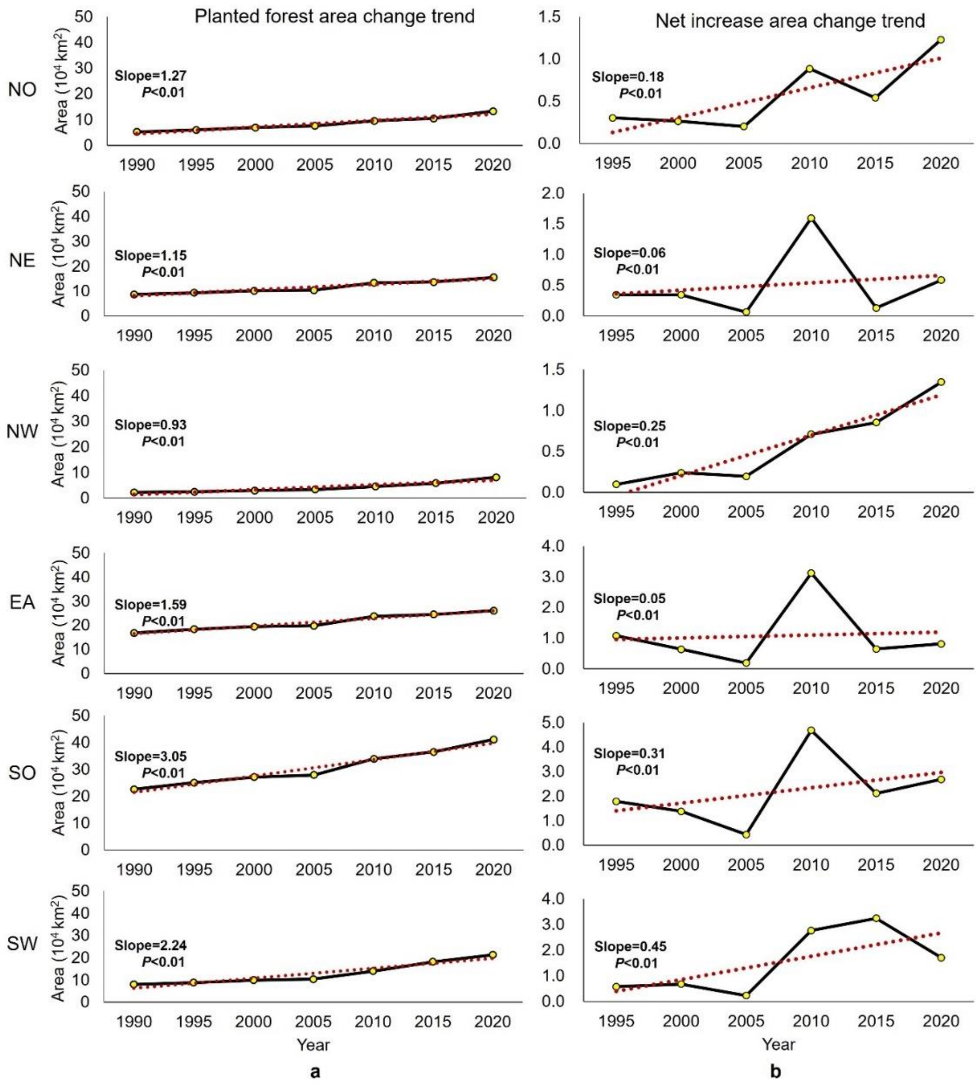

**Supplementary Fig. 7 | Spatial-temporal dynamics of planted forest area in China from 1990 to 2020. (a)** Spatial pattern of the increment trend in planted forest area at the national scale; **(b)** Spatial pattern of the net increase area trend at the national scale. Note: Dots indicate area changes that are statistically significant (t-test, $P < 0.05$) in more than half of 50 * 50 1-km$^2$ pixels. NE: northeast, NO: north, NW: northwest, EA: east, SO: south, SW: southwest.

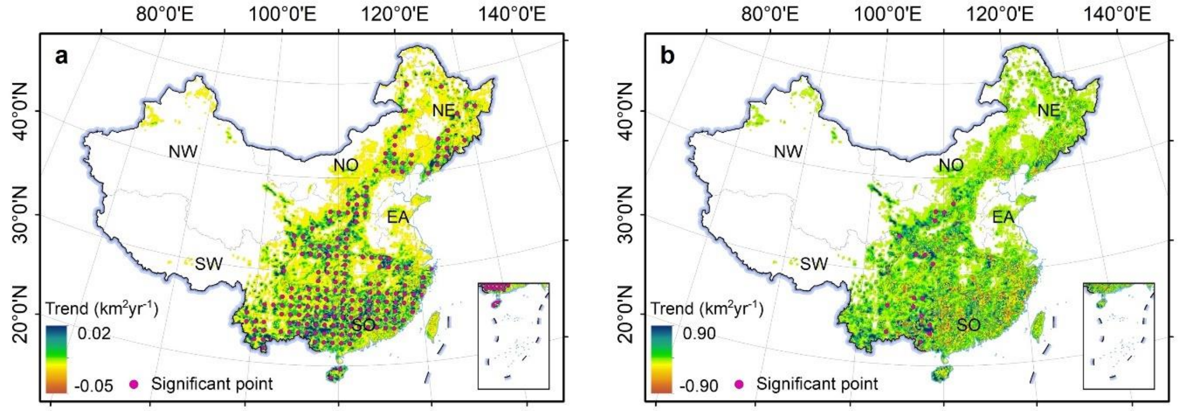

**Supplementary Fig. 8 | Main forestry engineering programs of China (from https://www.resdc.cn/). (a)** Eight forestry engineering regions in China; **(b)** Planned area of each of the eight forestry engineering programs; **(c)** Annual newly-formed forest area based on afforestation and reforestation from 1990 to 2020. SDP-TN: the Three-North Shelterbelt Development Program (1978-2050); SPLR: the Shelterbelt Program for Liaohe River (1993-2005); APTM: the Afforestation Program for Taihang Mountain (1986-2050); SPMRY: the Shelterbelt Program for Middle Reaches of Yellow River (1993-2010); SPHRTL: the Shelterbelt Program for Huaihe River and Taihu Lake (1993-); SDP-FR: the Shelterbelt Development Program in Five Regions including the Middle and Lower Reaches of the Yangtze River(1988-); SPPR: the Shelterbelt Program for Pearl River (1993-); CSP: the Coastal Shelterbelt Program (1987-2010).

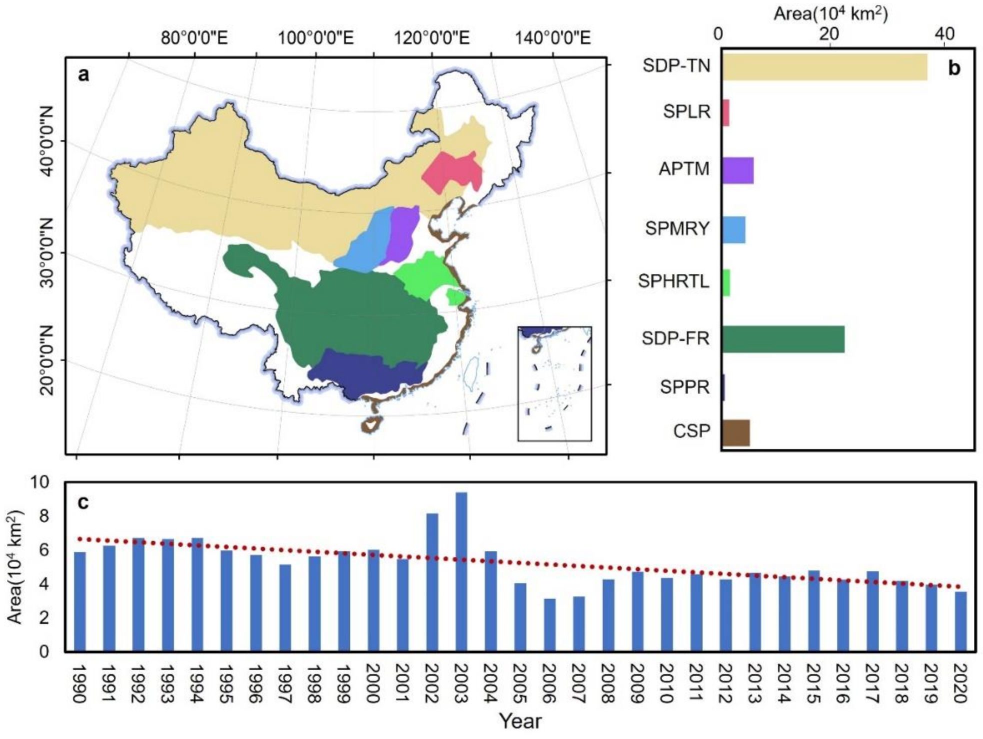

**Supplementary Fig. 9 | Spatial distribution of change frequency in planted forest area.**

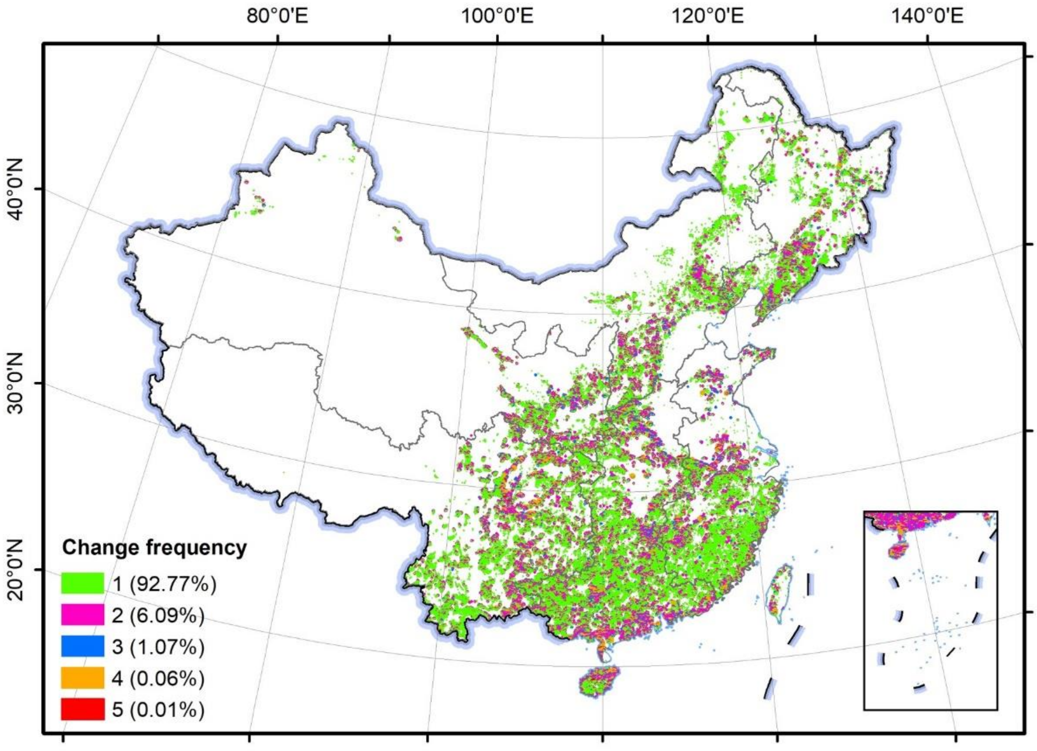

**Supplementary Fig. 10 | Change types of planted forest from 1990 to 2020. (a)** Transfer types of their composition in multi-change event (MCE) and single-change event (SCE); **(b)** Composition of change events in different regions. NE: northeast, NO: north, NW: northwest, EA: east, SO: south, SW: southwest.

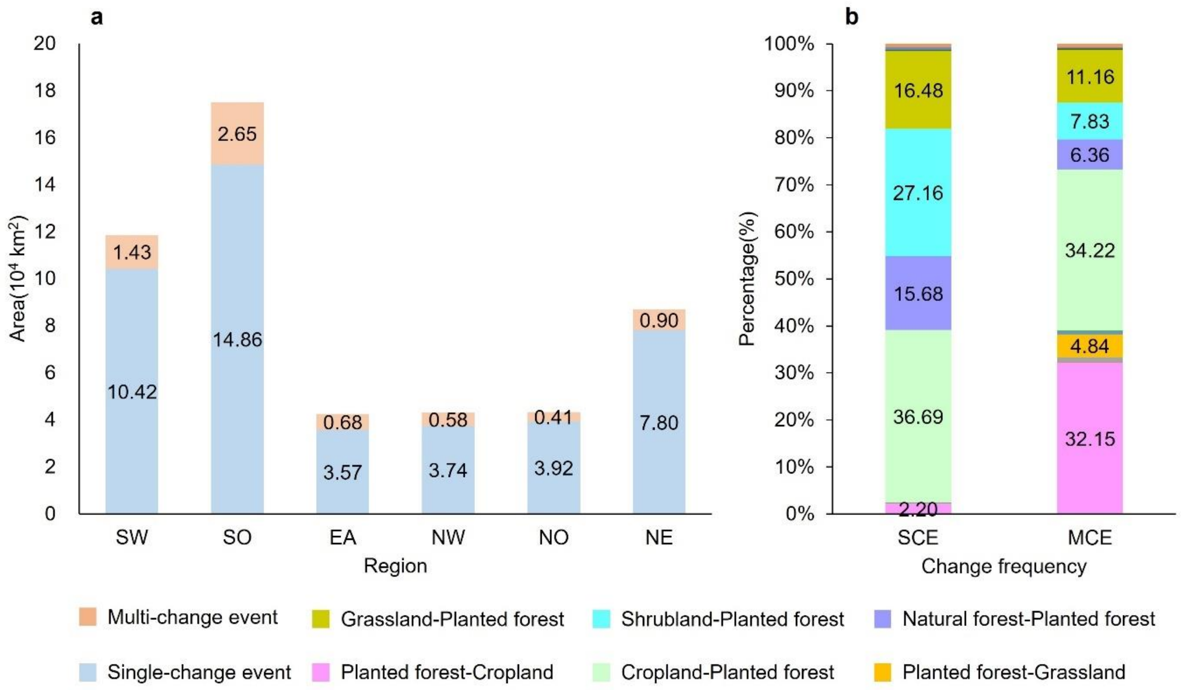

**Supplementary Fig. 11 | Distributions of training sample and testing sample. (a)** training samples; **(b)** the testing samples used in this study. NE, NO, NW, EA, SO, and SW represent the geographical divisions of northeast, north, northwest, east, south, and southwest in China, respectively.

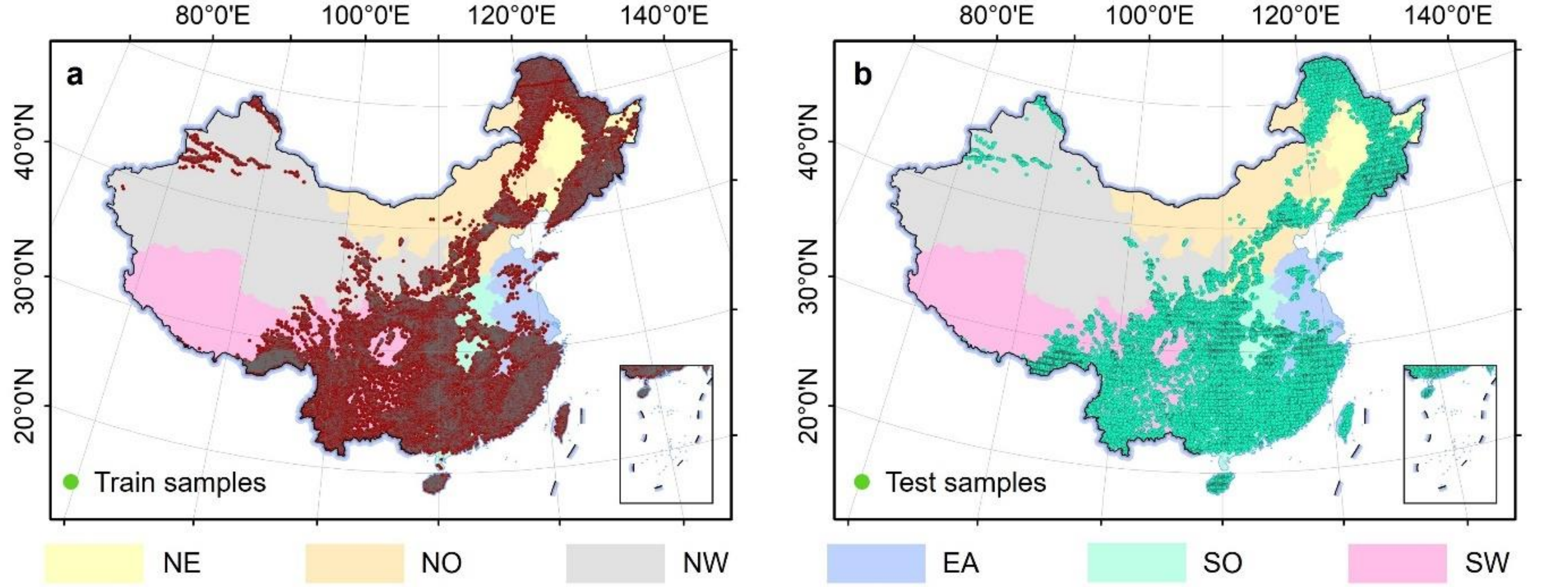

**Supplementary Fig. 12 | Feature selection using the RFE-CV approach in different regions during different periods**

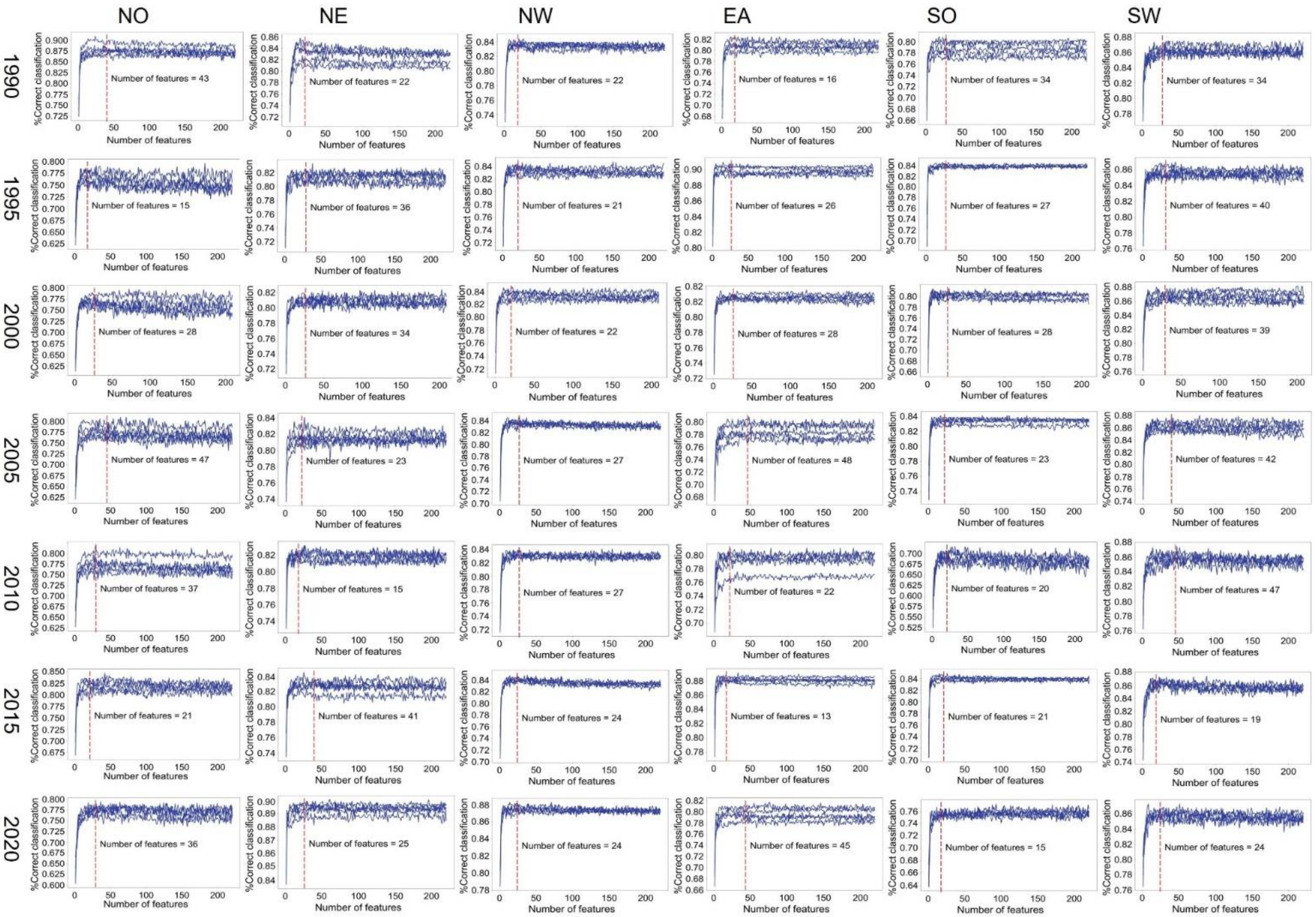

**Supplementary Fig. 13 | Distribution of validation samples collected by systematic sampling.**

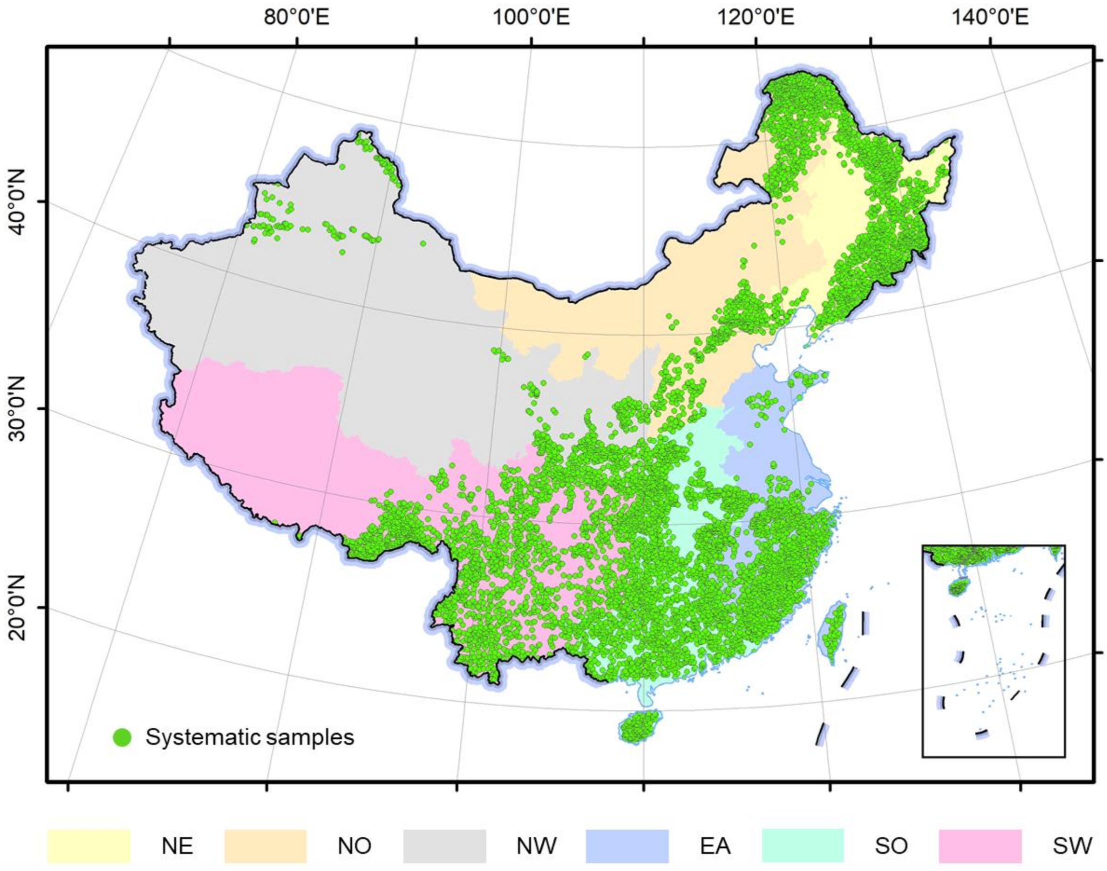

**Supplementary Fig. 14 | Carbon comparison with field surveys (a), Yang's aboveground biomass map (b), and Global aboveground biomass data released by Climate Change Initiative (CCI) Programme (c) at a 0.1° aggregated grid.**

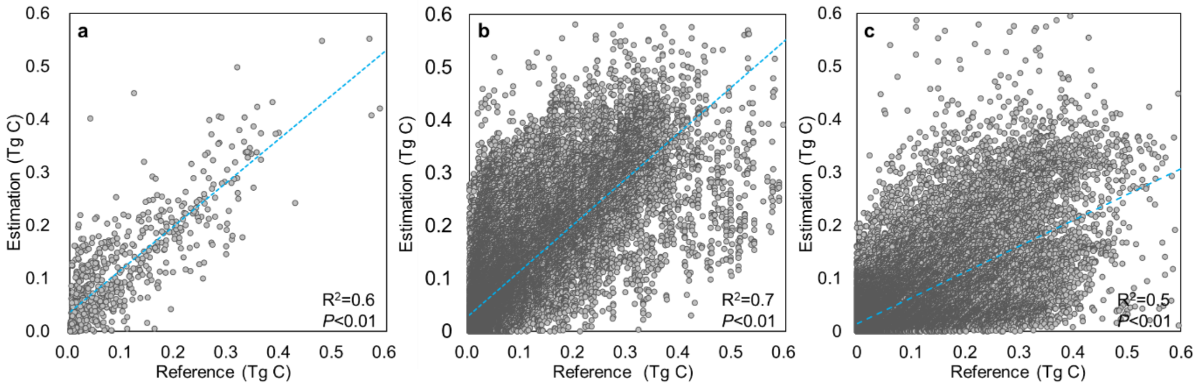

**Supplementary Fig. 15 | The composited images and their gaps for 1990, 1995, 2000, 2005, 2010, 2015, and 2020.**

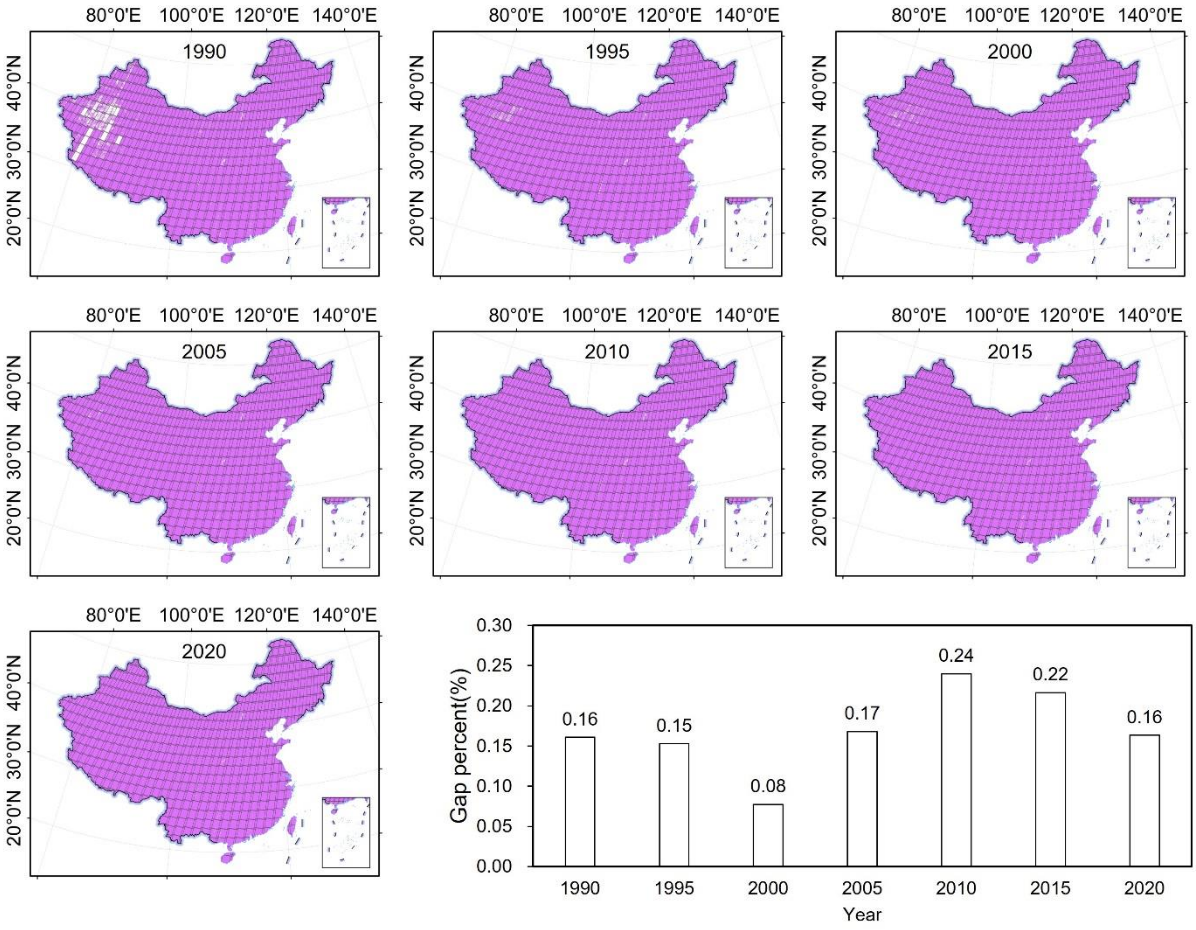

**Supplementary Fig. 16 | Distribution of pixel uncertainties for mapping planted forest.**

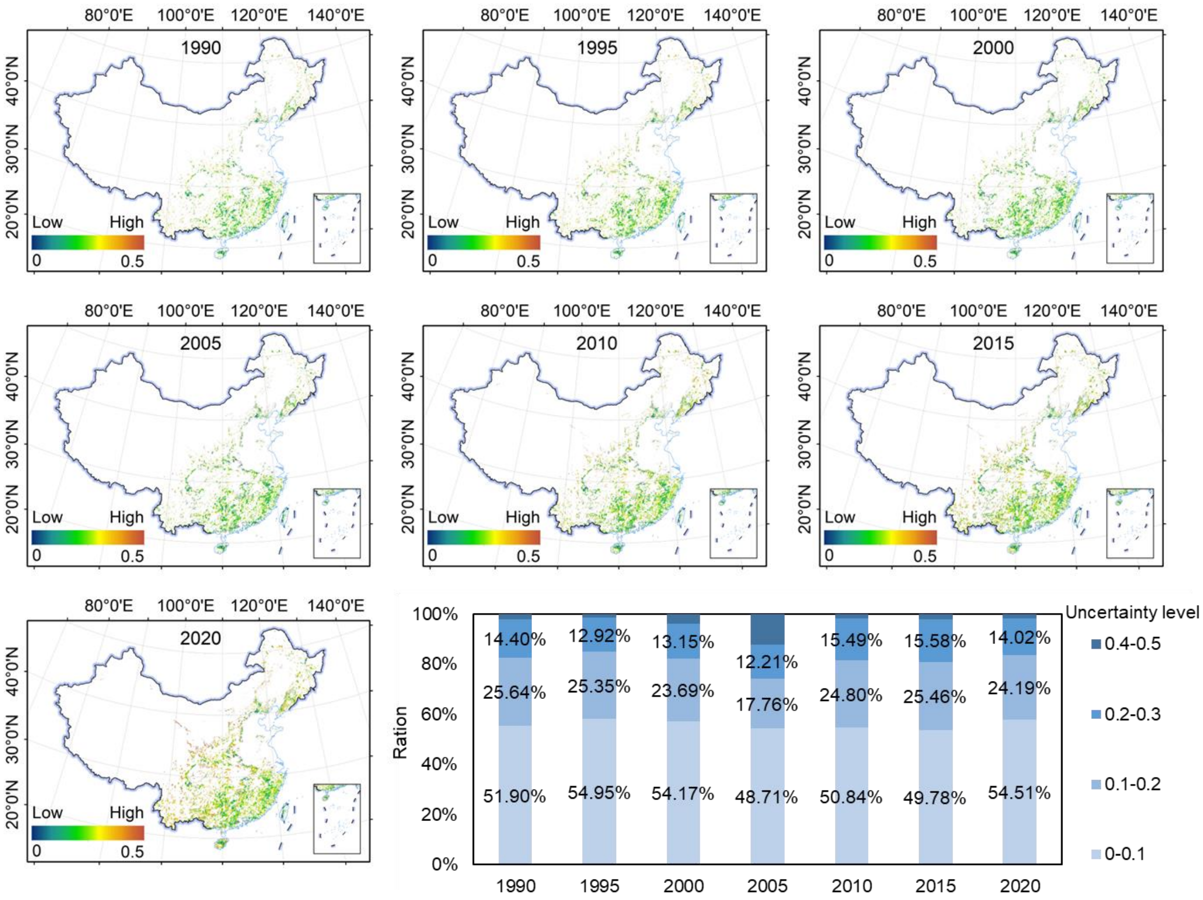

**Supplementary Fig. 17 | Discrepancies in carbon storage estimates caused by differences in vegetation types.**

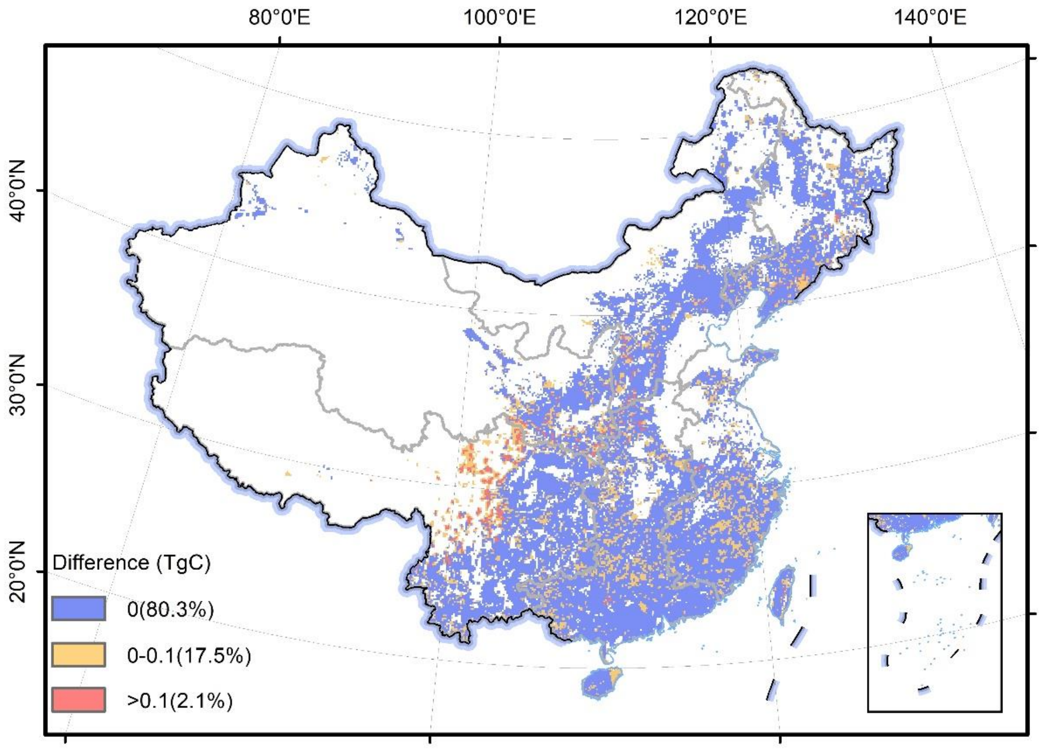

**Supplementary Fig. 18 | Forest regions of GLC_FCS30, CLCD, and CLUD dataset in 1990, 1995, 2000, 2005, 2010, 2015 and 2020.** GLC_FCS30 LULC data were downloaded from the Data Sharing and Service Portal (available at https://data.casearth.cn/en/) . CLCD LULC data were downloaded from https://zenodo.org/record/5816591#.Y9tkpnBBztU. CLUD LULC data were collected from the Resource and Environment Science and Data Center (available at https://www.resdc.cn/).

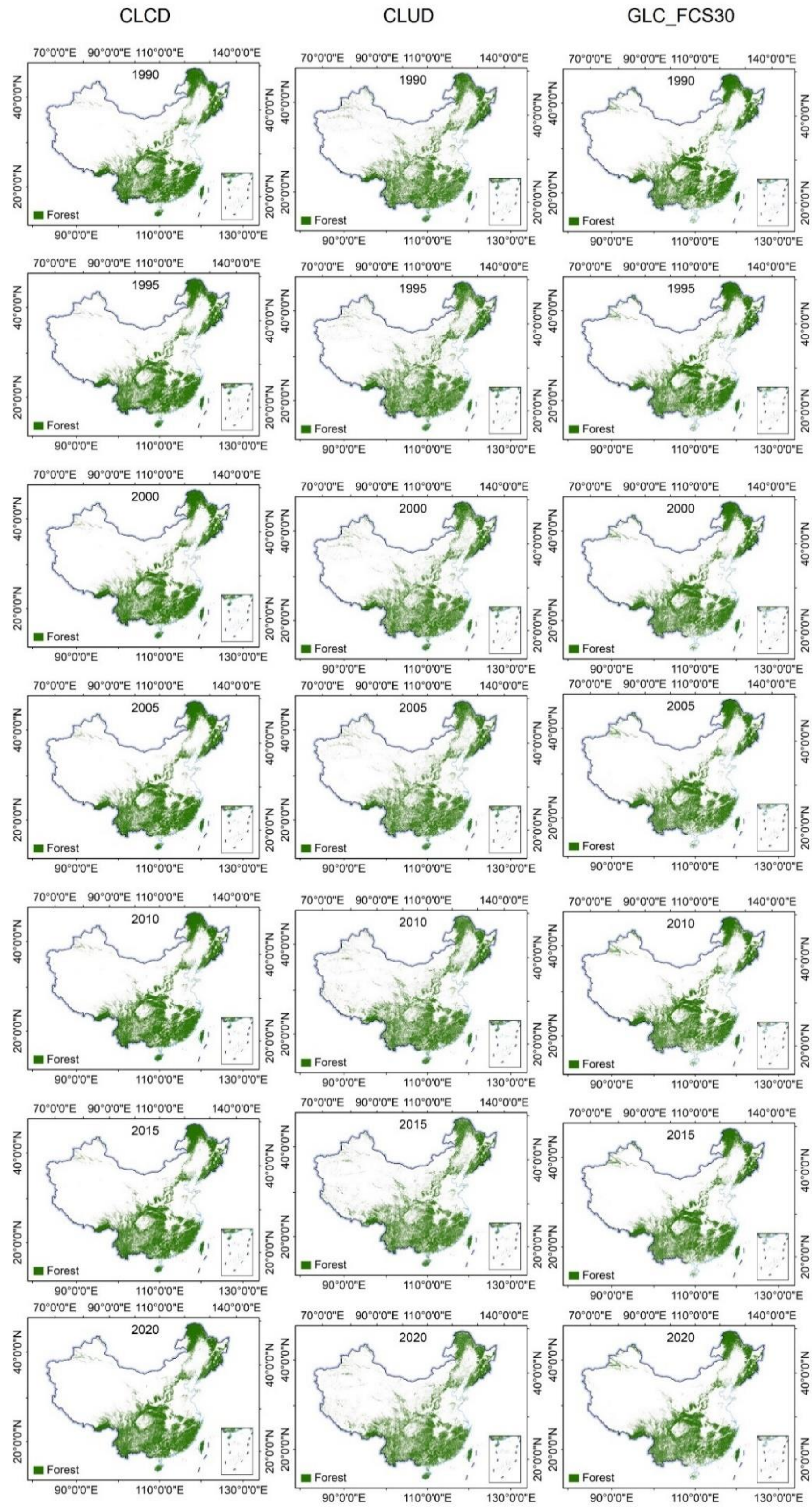

CLCD
CLUD
GLC_FCS30
1990
1995
2000
2005
2010
2015
2020
Forest
70°0'0"E
90°0'0"E
110°0'0"E
140°0'0"E
130°0'0"E
40°0'0"N
20°0'0"N

**Supplementary Fig. 19 | Comparison of overall accuracies between GLC-FCS30, CLCD, CLUD LULC datasets in the periods of 1990, 1995, 2000, 2005, 2010, 2015, and 2020.**

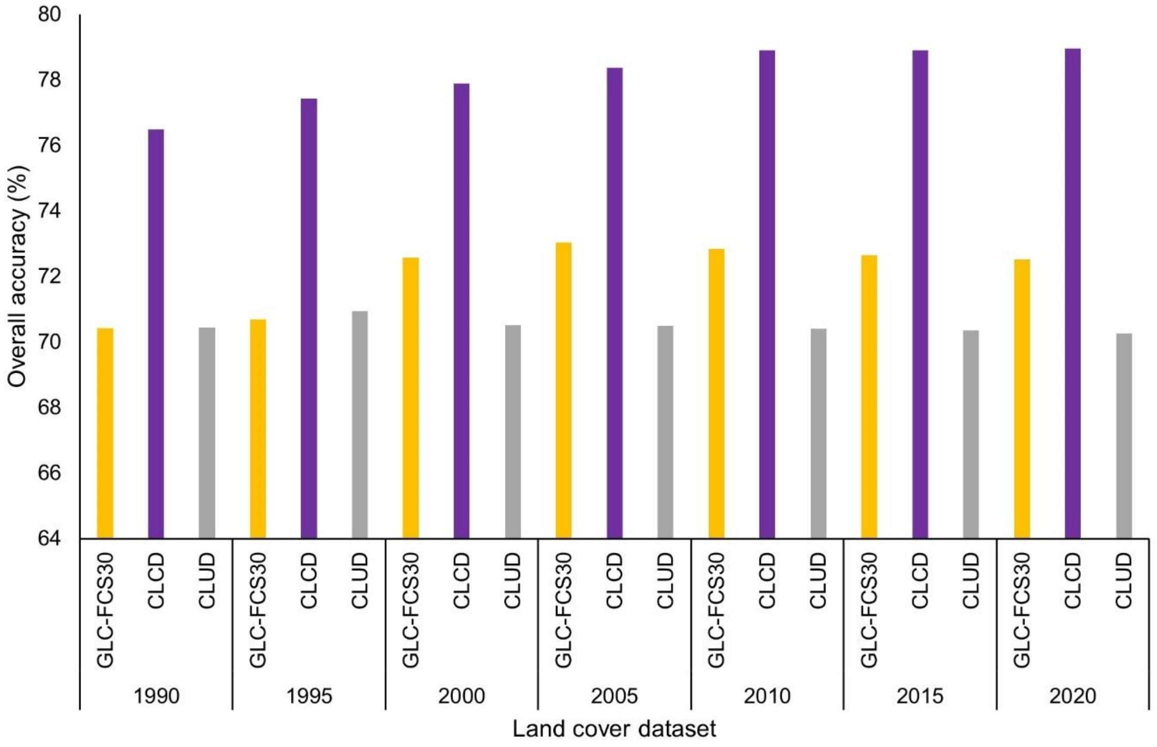

**Supplementary Table 1 | C storage estimated for 1990, 1995, 2000, 2005, 2010, 2015, and 2020.**

| Year | Carbon | SD |
|---|---|---|
| 1990 | 675.6 | 12.5 |
| 1995 | 760.4 | 12.3 |
| 2000 | 853.1 | 13.0 |
| 2005 | 977.9 | 13.8 |
| 2010 | 1343.7 | 14.4 |
| 2015 | 1559.9 | 15.1 |
| 2020 | 1873.1 | 16.2 |

**Supplementary Table 2 | Changes in C storage caused by conversion of other LULC to planted forests at the regional scale.**

| | Cropland (Tg) | | Forest (Tg) | | Shrub (Tg) | | Grassland (Tg) | | Other (Tg) | |
|---|---|---|---|---|---|---|---|---|---|---|
| Year | Carbon | SD | Carbon | SD | Carbon | SD | Carbon | SD | Carbon | SD |
| 1995 | 15.4 | 0.3 | 17.9 | 0.5 | 21.2 | 0.4 | 13.2 | 0.3 | 1.3 | 0.2 |
| 2000 | 12.8 | 0.3 | 13.2 | 0.4 | 20.1 | 0.4 | 11.0 | 0.3 | 1.0 | 0.1 |
| 2005 | 15.7 | 0.2 | 0.0 | 0.0 | 0.7 | 0.0 | 4.8 | 0.1 | 0.3 | 0.2 |
| 2010 | 67.8 | 2.5 | 87.2 | 3.5 | 36.4 | 1.5 | 48.4 | 1.1 | 2.0 | 0.3 |
| 2015 | 3.0 | 0.1 | 3.0 | 0.1 | 94.4 | 1.7 | 18.8 | 0.3 | 3.7 | 0.2 |
| 2020 | 77.0 | 0.8 | 0.3 | 0.0 | 3.6 | 0.1 | 39.7 | 1.5 | 3.2 | 0.1 |

**Supplementary Table 3| C storage caused by conversion of other LULC to planted forests at the regional scale.**

| Regions | Cropland | SD | Shrubland | SD | Grassland | SD | Natural forest | SD | Other | SD |
|---|---|---|---|---|---|---|---|---|---|---|
| North west | 10.60 | 0.20 | 11.10 | 0.50 | 32.10 | 1.60 | 4.60 | 0.30 | 0.22 | 0.10 |
| North | 6.10 | 0.30 | 10.80 | 0.70 | 27.10 | 0.60 | 9.50 | 2.80 | 1.32 | 0.30 |
| Northeast | 28.30 | 2.20 | 4.60 | 0.80 | 3.80 | 0.30 | 19.70 | 1.40 | 1.70 | 0.20 |
| East | 32.60 | 0.70 | 22.70 | 0.70 | 14.80 | 0.40 | 35.40 | 1.00 | 2.35 | 0.10 |
| South | 70.80 | 1.10 | 67.00 | 2.00 | 23.90 | 1.00 | 39.00 | 1.10 | 5.00 | 0.20 |
| South west | 43.30 | 0.60 | 60.20 | 0.80 | 34.10 | 0.60 | 13.50 | 0.60 | 0.96 | 0.10 |

**Supplementary Table 4 | Number and types of images used for planted forest mapping in 1990, 1995, 2000, 2005, 2010, 2015, and 2020.**

| Year | Landsat-4 | Landsat-5 | Landsat-7 | Landsat-8 | Landsat-9 |
|---|---|---|---|---|---|
| 1990 | 1,186 | 32,754 | -- | -- | -- |
| 1995 | -- | 35,446 | -- | -- | -- |
| 2000 | -- | 31,510 | 23,251 | -- | -- |
| 2005 | -- | 31,606 | 42,353 | -- | -- |
| 2010 | -- | 29,514 | 43,924 | -- | -- |
| 2015 | -- | -- | 49,623 | 49,267 | -- |
| 2020 | -- | -- | 33,494 | 42,219 | 1,601 |
| Sum | 1,186 | 160,830 | 192,645 | 91,468 | 1,601 |

**Supplementary Table 5 | Carbon density for various forest types from 1990 to 2020, CD is carbon density, CSD is standard deviation of carbon density. AddCD and AddCSD are carbon density and corresponding standard deviation of newly added planted forests during 2005-2010 and 2005-2020**

| Forest Type | CD1990 | CSD1990 | CD1995 | CSD1995 | CD2000 | CSD2000 | CD2005 | CSD2005 | CD2010 | CSD2010 | CD2015 | CSD2015 | CDOld2020 | CSDOld2020 | AddCD | AddCSD |
|---|---|---|---|---|---|---|---|---|---|---|---|---|---|---|---|---|
| Cold-temperate and temperate mountains needleleaf forest | 42.7 | 31.8 | 44.3 | 31.2 | 47.3 | 30.5 | 51.9 | 30.6 | 57.2 | 28.7 | 61.4 | 28.5 | 65.1 | 28.3 | 21.6 | 11.7 |
| Subtropical broadleaf deciduous forest | 19.3 | 13.5 | 19.8 | 13.8 | 21.4 | 15.1 | 24.9 | 17.2 | 29.9 | 18.4 | 34 | 19.9 | 38 | 21.4 | 17.1 | 6.8 |
| Subtropical mixed broadleaf evergreen and deciduous forest | 22.1 | 17.8 | 22.9 | 17.3 | 22.9 | 16.7 | 28 | 17.6 | 33.9 | 19.1 | 38 | 20 | 42.2 | 21 | 18.6 | 7 |
| Subtropical broadleaf evergreen forest | 20.7 | 15.8 | 21.6 | 15.2 | 22.1 | 15 | 26.2 | 15.8 | 31.1 | 16.6 | 34.9 | 17.1 | 38.4 | 17.6 | 17.5 | 5.4 |
| Subtropical monsoon broadleaf evergreen forest | 34.2 | 31.8 | 31.6 | 19.9 | 28.7 | 27.8 | 31.3 | 26.4 | 36.9 | 26.9 | 41.2 | 27.2 | 45.1 | 27.7 | 16.9 | 7.2 |
| Subtropical broadleaf evergreen sclerophyllous forest | 74.2 | 75.3 | 77.9 | 41.9 | 81.3 | 40 | 85.6 | 38 | 88 | 37.2 | 89.6 | 36.8 | 92.3 | 38.9 | 17.5 | 5.4 |
| Tropical monsoon rain forest | 20 | 8.8 | 20.8 | 10.1 | 20.6 | 10.7 | 22.3 | 11.8 | 28.6 | 12.6 | 32.9 | 13.4 | 37.1 | 14 | 15.8 | 5.6 |
| Tropical rain forest | 23.4 | 11.6 | 24.3 | 12.6 | 25.2 | 13.4 | 28.1 | 14.8 | 34.3 | 15.3 | 39.2 | 15.9 | 44.1 | 16.1 | 15.4 | 4.7 |

| | | | | | | | | | | | | | | | | |
|---|---|---|---|---|---|---|---|---|---|---|---|---|---|---|---|---|
| Subtropical, tropical bamboo forest and scrub | 19 | 15 | 19.9 | 16.3 | 21 | 13.5 | 26.1 | 15.3 | 30.6 | 16.4 | 33.9 | 17 | 37.1 | 17.7 | 17.8 | 6.2 |
| Temperate needleleaf forest | 23.9 | 18.3 | 24.6 | 18.8 | 26 | 19 | 29.4 | 18.8 | 35.7 | 18.5 | 39 | 18.3 | 42.2 | 18.1 | 15.5 | 6.1 |
| Subtropical needleleaf forest | 17.6 | 10.7 | 18 | 10.1 | 19.3 | 10.2 | 23.6 | 11.4 | 28.1 | 12.5 | 31.5 | 13.5 | 34.6 | 14.5 | 18.1 | 5.9 |
| Tropical needleleaf forest | 17.6 | 10.7 | 18 | 10.1 | 19.3 | 10.2 | 23.6 | 11.4 | 28.1 | 12.5 | 31.5 | 13.5 | 34.6 | 14.5 | 18.1 | 5.9 |
| Subtropical and tropical mountains needleleaf forest | 81.1 | 51.1 | 79 | 51.7 | 79.2 | 51.3 | 81.2 | 50.5 | 85.1 | 48.9 | 87.8 | 48.3 | 90.3 | 47.6 | 28.9 | 5.3 |
| Temperate mixed needleleaf and broadleaf deciduous forest | 82.3 | 40 | 78.7 | 42.2 | 82.3 | 40.5 | 83.2 | 39.5 | 87.2 | 36.5 | 90.4 | 34.5 | 93 | 33 | 25 | 7.1 |
| Subtropical mountains mixed needleleaf, broadleaf evergreen and deciduous forest | 47.4 | 43 | 41.2 | 38.3 | 40.1 | 33.8 | 46.8 | 32.5 | 54.4 | 32.7 | 58.4 | 31.7 | 62 | 30.7 | 29.8 | 5.5 |
| Temperate broadleaf deciduous forest | 40.3 | 34.5 | 41 | 34 | 42.4 | 33.5 | 45.6 | 33 | 53.5 | 33.3 | 57.7 | 32.7 | 61.4 | 31.9 | 19.8 | 10.1 |
| Temperate microphyllous deciduous woodland | 40.3 | 34.5 | 41 | 34 | 42.4 | 33.5 | 45.6 | 33 | 53.5 | 33.3 | 57.7 | 32.7 | 61.4 | 31.9 | 19.8 | 10.1 |

# Reference


1. Zhang, X. et al. GLC_FCS30: global land-cover product with fine classification system at 30 m using time-series Landsat imagery. *Earth Syst. Sci. Data* **13**, 2753-2776 (2021).
2. Yang, J. & Huang, X. The 30m annual land cover dataset and its dynamics in China from 1990 to 2019. *Earth Syst. Sci. Data* **13**, 3907-3925 (2021).
3. Zhou, J., Jia, L. & Menenti, M. Reconstruction of global MODIS NDVI time series: Performance of Harmonic ANalysis of Time Series (HANTS). *Remote Sens Environ*. **163**, 217-228 (2015).
4. Juola, J., Hovi, A. & Rautiainen, M. Classification of tree species based on hyperspectral reflectance images of stem bark. *Eur. J. Remote. Sens.* **56**, 1-15 (2022).
5. Pandey, S., Miri, R., Sinha, G. R. & Raja, R. AFD filter and E2N2 classifier for improving visualization of crop image and crop classification in remote sensing image. *Int J Remote Sens.* **43**, 5848-5873 (2022).
6. Matarira, D., Mutanga, O. & Naidu, M. Google Earth Engine for Informal Settlement Mapping: A Random Forest Classification Using Spectral and Textural Information. *Remote Sens.* **14(20)**, 5130 (2022).
7. Gorelick, N. et al. Google Earth Engine: Planetary-scale geospatial analysis for everyone. *Remote Sen Environ*. **202**, 18-27 (2017).
8. Cheng, K. et al. Mapping China's planted forests using high resolution imagery and massive amounts of crowdsourced samples. *ISPRS J. Photogramm*. **196**, 356-371 (2023).
9. Wang, J. et al. Mapping sugarcane plantation dynamics in Guangxi, China, by time series Sentinel-1, Sentinel-2 and Landsat images. *Remote Sens Environ*. **247**, 111951 (2020).
10. Fagan, M. E. et al. Mapping pine plantations in the southeastern U.S. using structural, spectral, and temporal remote sensing data. *Remote Sens Environ*. **216**, 415-426 (2018).
11. Bauer, E. & Kohavi, R. An Empirical Comparison of Voting Classification Algorithms: Bagging, Boosting, and Variants. *Mach Learn*. **36**, 105-139 (1999).
12. Cheng, K., Wang, J. & Yan, X. Mapping Forest Types in China with 10 m Resolution Based on Spectral–Spatial–Temporal Features. *Remote Sens.* **13**, 973 (2021).
13. Wang, X. et al. Rebound in China's coastal wetlands following conservation and restoration. *Nat. Sustain*. **4**, 1076-1083 (2021).
14. Rizayeva, A., Nita, M. D. & Radeloff, V. C. Large-area, 1964 land cover classifications of Corona spy satellite imagery for the Caucasus Mountains. *Remote Sens Environ.* **284**, 113343 (2023).
15. Peng, S. et al. Afforestation in China cools local land surface temperature. *Proc. Natl. Acad. Sci. U.S.A*. **111**, 2915-2919 (2014).
16. Calderón-Loor, M., Hadjikakou, M. & Bryan, B. A. High-resolution wall-to-wall land-cover mapping and land change assessment for Australia from 1985 to 2015. *Remote Sen Environ. 252*, 112148 (2021).
17. Loosvelt, L. et al. Random Forests as a tool for estimating uncertainty at pixel-level in SAR image classification. *Int. J. Appl. Earth Obs. Geoinformation* **19**, 173-184 (2012).
