# Supplementary material for: Carbon storage through China’s planted forest expansion
Source: Nat Commun. 2024 May 15;15:4106. doi: 10.1038/s41467-024-48546-0 (PMC11096308; doi:10.1038/s41467-024-48546-0)
Supplement: Supplementary file 3 — Description of Additional Supplementary Files [file 41467_2024_48546_MOESM3_ESM.pdf]

Description of Additional Supplementary Files

Supplementary Data 1: Feature set for mapping planted forest for different regions in different periods. The grey background in the table represents the features selected for mapping planted forests using the RFE-CV approach for the region from 1990 to 2020.
